# Distrusting minds, skeptical judgments? No evidence for a trust-truth link

**DOI:** 10.3389/fpsyg.2025.1626047

**Published:** 2025-09-30

**Authors:** Anna-Marie Bertram, Fanny Lalot, Lisa van der Werff, Rainer Greifeneder

**Affiliations:** ^1^Center of Social Psychology, Faculty of Psychology, University of Basel, Basel, Switzerland; ^2^DCU Business School, Irish Institute of Digital Business, Dublin City University, Dublin, Ireland

**Keywords:** trust propensity, social trust, truth judgments, interpersonal trust, generalized trust

## Abstract

**Introduction:**

Many daily situations require rapid judgments about whether information is true or false based on limited information. Prior research has predominantly examined how statement and source characteristics influence these judgments. The present study shifts its focus to individuals’ stable traits, specifically trust propensity, and its role in shaping truth judgments.

**Methods:**

Across three studies considering different social contexts (*N* = 679), we investigate whether trust propensity (i.e., one’s general tendency to trust others) and the closely related construct of social trust (i.e., one’s perception of others in general as more or less trustworthy) are associated with a greater likelihood of judging various statements as true.

**Results:**

Contrary to expectations, linear mixed-effects analyses indicated that neither trust propensity nor social trust had any significant relationship with truth judgments across contexts. Bayesian analyses further indicated strong overall support for the null hypothesis over the alternative.

**Discussion:**

Thus, this research highlights that, contrary to common-sense belief, trust-related traits may play a relatively minor role in decision-making under uncertainty.

## Introduction

Every day, individuals are confronted with statements whose truth they cannot directly verify—be it while reading the headlines of a newspaper, listening to a friend make a bold claim, or watching a political debate. Despite this uncertainty, they constantly need to decide: Do I believe this or not? While these so-called truth judgments have been studied extensively, research has largely focused either on characteristics of the message itself or on characteristics of the source, and relatively less attention has been paid to the role of stable individual dispositions.

According to Rotter’s (1971) influential paper, to trust someone means to rely on their word or promise, implying an inherent belief in the truth of what this person says. Rotter goes further by stating that “implicit in all these situations is the problem of whether or not to believe the other person. On this basis, we have hypothesized a generalized expectancy of trust or distrust” (p. 445). This statement appears highly intuitive and consistent with common lay beliefs: people who are overly trusting should be more likely to believe statements to be true, while people who are skeptical should be more likely to disagree with statements for which they do not know the correct answer. Despite the apparent plausibility of this lay belief, to the best of our knowledge, to date, no experimental designs have systematically assessed the relationship between trust propensity and randomly varied truth judgments.

The present study aims to advance the empirical understanding of how individual dispositions, specifically *trust propensity*, may shape truth judgments. While trust propensity has been linked to various social behaviors—such as cooperation (Colquitt et al., 2007)—its impact on how people assess the truthfulness of statements has not been systematically investigated. In the following sections, we describe existing studies on trust propensity and the neighboring construct of social trust and develop the hypothesis that we would expect them to be related to truth judgments. We then present the results of three experiments (*N* = 679) that investigate these relationships.

### Generalized beliefs about others: trust propensity and social trust

Trust propensity is a stable trait that reflects a “general willingness to trust others, regardless of social and relationship-specific information” (Frazier et al., 2013, p. 77). It is conceptualized as a stable individual trait that is largely invariant across different trust situations. Trust propensity plays a crucial role, particularly in the early phases of a relationship when information about the trustee’s trustworthiness may be lacking. Its impact then decreases (Dietz and Den Hartog, 2006) but does not disappear (Colquitt et al., 2007) as the relationship progresses. Trust propensity is thus conceptualized as a characteristic of the trustor, which complements characteristics of the situation/relationship and of the trustee (e.g., dimensions of trustworthiness) in different integrative models of trust (e.g., Mayer et al., 1995; Schoorman et al., 2007; McAllister, 1995).

The role of trust propensity in interpersonal interactions has been demonstrated in numerous studies. For instance, individuals with low trust propensity tend to respond to uncertain situations with caution or even negativity, often without a specific reason to justify these feelings (Falcone and Castelfranchi, 2001; Graziano and Tobin, 2017). These differences are particularly evident in early-stage relationships or situations perceived as subjectively important, where individuals with high trust propensity are more inclined to share personal information and expect reciprocity (Ferguson and Peterson, 2015). Conversely, low-trust individuals tend to withhold resources, minimize dependence, and approach social situations with increased caution.

Trust propensity is closely related to, albeit conceptually distinct from, social trust, which refers to a generalized belief that people are inherently trustworthy and will act in ways that promote social cohesion while minimizing harm to others (Delhey and Newton, 2005). In McKnight and Chervany’s (2001) trust model, for example, social trust aligns with the dimension of “faith in humanity,” which reflects a generalized belief in others’ competence, benevolence, and integrity. While trust propensity reflects a dispositional inclination to extend trust, capturing how willing an individual is generally to rely on others, social trust reflects a broader orientation toward society, encompassing expectations about the trustworthiness of people in general.

Both constructs share the idea that trust involves a readiness to accept vulnerability in social interactions, yet they differ in their emphasis: trust propensity is more rooted in one’s own generalized tendency to approach others with trust, whereas social trust centers on beliefs about whether others, as a collective, are likely to be honest and fair. Zhang (2021, p. 1) described a similar distinction between “an individual’s propensity to trust (one’s ‘trustingness’ or the extent to which one feels able to trust others) and their other-focused trust (the extent to which one feels that others are worthy of one’s trust).” Both trust propensity and social trust shape the way individuals navigate everyday social interactions—particularly in situations where they are confronted with information from others. In these moments, people must often make implicit judgments about whether to believe what is being said. Such trust-related tendencies influence how individuals interact with and interpret social information, particularly in uncertain situations. One conceptual framework that captures these evaluative processes is that of *truth judgments*.

### Truth judgments

#### What shapes truth judgments?

Truth judgments pertain to individuals’ subjective evaluations of whether a statement or claim is true or false (Brashier and Marsh, 2020). These judgments are shaped by multiple factors that can be categorized into three broad domains: characteristics of the *statement* itself, characteristics of the *source* of the statement, and attributes of the *individual* making the judgment.

First, the perceived truthfulness of a statement depends on its specific characteristics. Research demonstrates that even subtle changes in framing can significantly alter truth judgments. For instance, negatively framed statements (e.g., “61 percent of German women are unsatisfied with their appearance”) are consistently judged as more likely to be true than their positively framed counterparts (e.g., “39 percent of German women are satisfied with their appearance”; Jaffé and Greifeneder, 2021).

Perceived truthfulness is further influenced by various characteristics of the source. For instance, statements attributed to experts or high-credibility sources are consistently judged as more truthful compared to identical claims from less credible sources (Pornpitakpan, 2004).

In addition to the characteristics of the statement and the source, individual factors also come into play. People naturally favor statements that align with their existing beliefs, consistent with an extensive literature on motivated reasoning (Fazio and Sherry, 2020). Memory processes also play a key role, notably through the well-documented illusory truth effect, which describes how repeated or familiar statements are more likely to be judged as true regardless of their actual veracity (Unkelbach and Rom, 2017). Processing fluency, or the subjective ease with which information is processed, represents another important mechanism. Fluency can arise from features of the statement itself, such as font clarity or syntactic simplicity, but is ultimately experienced as a property of the perceiver’s cognitive processing. Statements that feel easier to read, comprehend, or recall are typically judged as more truthful (Reber and Schwarz, 1999). Importantly, this fluency effect operates independently from emotional influences, where affective states may bias truth judgments through different pathways (Dunn and Schweitzer, 2005).

More crucially for our present purpose, base rates—people’s mental representations of the general distribution of true and false statements—serve as a cognitive bias that influences truth judgments even before a statement is presented, depending on the situation and source (Brashier and Marsh, 2020). Base rate beliefs emerge from an internalized sense of knowledge or intuition, allowing individuals to make assumptions about various distributions in the world around them and derive factual judgments accordingly. This cognitive bias can shape truth judgments in systematic ways. For instance, if someone assumes that photographs always depict reality, they are much less likely to question a specific photograph’s truthfulness. In such cases, a base rate bias fosters a tendency to perceive certain types of information as more likely to be true.

#### From generalized trust to truth judgment

An emerging literature has endeavored to examine whether generalized trust influences deception detection accuracy or systematic biases in truth judgment. Early theorizing (Carter and Weber, 2010) suggested that highly trusting individuals might be either more gullible or better lie detectors because of greater exposure to deception. More recent large-scale replication efforts, however, have not supported this view. Levine et al. (2024), for example, found no relationship between generalized trust and deception detection accuracy. Instead, their findings supported the truth-default theory (Levine, 2024), showing that generalized trust is only associated with increased truth bias. The truth bias is the tendency to believe others regardless of actual veracity (Levine et al., 1999), which shifts the direction of errors without improving overall accuracy. This pattern has been described as the “veracity effect” (Levine, 2018b). Consistent with this interpretation, meta-analytic evidence shows that individual differences in lie detection accuracy are reliably very small (Bond and DePaulo, 2008), casting doubt on the claim that personality traits such as trust meaningfully increase deception detection.

Given these limitations, it becomes important to consider not whether people can detect lies more accurately, but how they generally approach the task of judging statements. Accordingly, our research shifts from deception detection accuracy to people’s intuitive tendencies in truth assessment. Trust propensity may bias individuals’ judgments toward accepting or rejecting statements as true, regardless of actual veracity. The present studies, therefore, examine whether dispositional trust systematically shapes truth judgments across different social contexts (e.g., courts, colleagues, friends), thereby clarifying the extent to which personality-based trust propensities influence everyday veracity assessments.

### The present study

We argue that individuals with a higher trust propensity should be more likely to assume that others generally tell the truth, thus increasing their likelihood of rating any given statement as true. To test this idea, we conducted three preregistered studies investigating whether higher levels of trust propensity are associated with a greater likelihood of judging ambiguous statements as true. The studies consistently tested our general hypothesis while varying in contextual presentation: the first study presented the statements without any context, displayed as text on a blank background. The second study introduced social cues at different visual and descriptive levels. The third study embedded the statements within a courtroom context. In all studies, we additionally measured participants’ general tendency to believe that people tell the truth or lie (hereafter, *deception base rate belief*) to explore its relationship to trust propensity, social trust, and truth judgments.

For each study described below, we specify how we determined our sample size, all data exclusions, and all measures. Materials, data, and code for the analyses are publicly available on the OSF: https://osf.io/st94x/?view_only=7dca49e7fc0a4d7bbe05365ad930f77c. Data were analyzed and visualized using *RStudio*, version 2024.12.0. Links to preregistration are included in the section for each study.

## Study 1

Study 1 provided a first test of the relationship between trust propensity and truth judgments and was designed as a two-wave questionnaire. The study design, materials, sample size, rules for exclusion, and hypothesis were preregistered: https://aspredicted.org/xg54-3gw5.pdf.

The roots of trust in another person can be understood as a trait, a state, or a complex interplay of both, and this distinction has been central to a number of conceptual frameworks. McKnight and Chervany (2001), for example, differentiated between faith in humanity, the belief that most people are well-meaning and dependable; a trusting stance, a general willingness to rely on others even in the absence of concrete information; and perceptions of the trustee’s benevolence, integrity, and competence. Similarly, McAllister (1995) conceptualized trust as grounded in both affect-based goodwill and cognition-based assessments of integrity and reliability.

The items we employed in our three studies to capture trust propensity and social trust (Table 1) can be further explained by mapping them onto these theoretical perspectives. Items from the European Social Survey (e.g., “Generally speaking, would you say that most people can be trusted or that you cannot be too careful in dealing with people?” “Would you say that most of the time people try to be helpful or that they are mostly just looking out for themselves?” and “Do you think most people would try to take advantage of you if they got a chance, or would they try to be fair?”), as well as Kelley et al. (2003; “There are only a few people I can trust completely”), primarily reflect faith in humanity/moral character, capturing expectations that others will act fairly and refrain from exploitation.

**Table 1:** Items measuring trust propensity/social trust in Study 1, source, and assigned scale as a result of the exploratory factor analysis.

Item	Source	Assigned scale
Generally speaking, would you say that most people can be trusted or that you cannot be too careful in dealing with people?	European Social Survey	Social Trust
Would you say that most of the time, people try to be helpful or that they are mostly just looking out for themselves?	European Social Survey	Social Trust
Do you think most people would try to take advantage of you if they got a chance, or would they try to be fair?	European Social Survey	Social Trust
How much do you trust people you know personally?	World Values Survey	Excluded from analysis
How much do you trust people you meet for the first time?	World Values Survey	Excluded from analysis
There are only a few people I can trust completely.	Kelley et al. (2003)	Social Trust
I see myself as someone who is generally trusting.	Kelley et al. (2003)	Trust Propensity
I usually trust people until they give me a reason not to trust them.	Frazier et al. (2013)	Trust Propensity
Trusting another person is not difficult for me.	Frazier et al. (2013)	Trust Propensity
My typical approach is to trust new acquaintances until they prove I should not trust them.	Frazier et al. (2013)	Trust Propensity
My tendency to trust others is high.	Frazier et al. (2013)	Trust Propensity

In contrast, the items adapted from Frazier et al. (2013) align more closely with a trusting stance/goodwill orientation, which reflects a dispositional readiness to extend trust in the absence of specific knowledge about the other party. For example, “I usually trust people until they give me a reason not to trust them,” “Trusting another person is not difficult for me,” and “My typical approach is to trust new acquaintances until they prove I should not trust them” emphasize a general willingness to rely on others without prior relationship cues. Similarly, “My tendency to trust others is high” (Frazier et al., 2013) and “I see myself as someone who is generally trusting” (Kelley et al., 2003) capture this broad orientation toward trusting others as part of one’s self-concept.

### Methods

#### Participants and design

Participants were recruited from the online crowdsourcing platform Prolific, with the requirements of being at least 18 years old and living in the UK. We determined a target sample size of 250 participants, taking into account past research, practical aspects, and feasibility. Schönbrodt and Perugini (2013) suggest that a sample size of 250 participants provides 80% power to detect a correlation as small as 0.10, with a width of the corridor of stability of 0.10. We oversampled by 20% to compensate for exclusions and potential loss of participants between the two measurement points (T1 and T2) and therefore recruited 300 participants at T1. Of those, 265 also completed T2 (i.e., ≈12% dropout between T1 and T2). There were 126 men and 139 women with a mean age of 42.48 years (*SD* = 12.89). No participant had to be excluded based on our preregistered exclusion criteria (which were: not currently living/having lived in the UK, incorrect responses to attention checks, low participation seriousness rating (<6 out of 9 points), voluntary withdrawal from data analysis, and self-reported language difficulties).

To avoid response bias, we assessed trust propensity and truth judgments at two separate time points. The first questionnaire (T1) provided a general introduction and assessed participants’ demographics before evaluating their trust propensity and base rate beliefs regarding deception. In the second questionnaire (T2), participants were asked to rate 37 short statements as true or false. They were finally debriefed, thanked, and remunerated.

#### Materials

**Trust Propensity/Social Trust (T1).** We used 11 items to measure trust propensity/social trust, adapted from validated questionnaires (see Table 1). All items were rated on a 7-point Likert scale (1 = Strongly disagree to 7 = Strongly agree).

**Deception Base Rate Belief (T1).** A slider scale ranging from 0 to 100 percent was used to assess deception base rate belief. The item was phrased as follows: “In life, people might try to lie to you if they get the chance, or they could try to be honest. What percentage of people do you expect to lie to you in any given interaction?” (*M* = 37.45, *SD* = 20.80).

**Truth Judgments (T2).** Participants evaluated 37 ambiguous statements (e.g., “Drinking alcohol decreases core body temperature”; all statements are reported in Supplementary Table SM1). These were selected based on a pretest (*N* = 50) that confirmed ambiguity in believability (mean ambiguity value: 0.40–0.60). For each statement, participants indicated on a dichotomous scale whether it was “most likely true” (coded 1) or “most likely false” (coded 0). The procedure followed the standard protocol established in previous research on truth judgments (e.g., Brashier and Marsh, 2020; Hilbig, 2012; Hilbig et al., 2015; Jaffé and Greifeneder, 2021), meaning statements were presented without contextual framing and in a randomized order. Participants judged an average of *M* = 19.08 (*SD* = 4.62) statements as true. The average truth ratings per statement ranged from *M* = 0.32 to *M* = 0.75. No statement was judged as either false or true by all participants.

### Results

#### Factor analysis on the trust propensity/social trust measure

As preregistered, we first conducted an exploratory factor analysis to assess whether the different items would reveal an organization into different factors or rather a general construct of generalized beliefs about others. We relied on a multiple-criteria approach to determine the number of factors to extract. We considered the following methods: sequential *χ*^2^ model tests (and the lower bound of RMSEA’s 90% confidence interval), revised parallel analysis, Hull’s method (based on CFI and RMSEA), Ruscio’s comparison data, and the empirical Kaiser criterion (see Auerswald and Moshagen, 2019).

Five of the 11 factor retention criteria consistently supported a two-factor solution, while three methods favored a single-factor structure. Three criteria indicated more complex solutions, with no clear consensus. To resolve this discrepancy, we conducted direct model comparisons between one- and two-factor solutions.

The two-factor model accounted for 64% of the cumulative variance and demonstrated a better fit across multiple indices: *χ*^2^(19) = 48.49, *p* < 0.001, *χ*^2^/df = 2.55, CFI = 0.965, RMSEA = 0.076, 90% CI [0.054, 0.104], SRMR = 0.033, Bayesian information criterion (BIC) = −57.53 (one-factor solution: *χ*^2^(27) = 175.38, *p* < 0.001, *χ*^2^/df = 6.50, CFI = 0.875, RMSEA = 0.144, TLI = 0.933, 90% CI [0.124, 0.165], SRMR = 0.072, BIC = 24.72; 55% of the cumulative variance explained), a difference which was statistically significant, Δ*χ*^2^(8) = 126.89, *p* < 0.001. We thus retained the two-factor model for further analysis. In this two-factor solution, four items loaded on the first factor and five items on the second. Two items had cross-loadings greater than 0.30 and were excluded, resulting in a final set of nine items.

The first factor (four items) may be best understood as capturing social trust, with items such as *“Generally speaking, would you say that most people can be trusted or that you cannot be too careful in dealing with people?”* emphasizing expectations about others rather than personal tendencies. The second factor (five items), in contrast, may be best understood as capturing trust propensity, with items focusing on the self, such as *“My tendency to trust others is high.”* We computed two separate mean scores for trust propensity (*M* = 4.66, *SD* = 1.34; *α* = 0.94) and social trust (*M* = 3.89, *SD* = 1.03; *α* = 0.77). The zero-order correlation between social trust and trust propensity was significant and positive, *r* = 0.64, 95% CI [0.57, 0.70], *p* < 0.001.

Social trust was negatively related to deception base rate belief, *r* = −0.58, *p* < 0.001, 95% CI [−0.65, −0.49], indicating that individuals who believed others are more likely to lie generally reported lower social trust. Trust propensity also showed a strong negative correlation with deception base rate belief, *r* = −0.52, *p* < 0.001, 95% CI [−0.60, −0.43].

#### Testing the relationship between trust propensity/social trust and truth judgments

**Preregistered analysis.** We examined the relationship between trust propensity/social trust and truth judgments using mixed-effects models (binomial distribution; testing one predictor at a time). Contrary to our hypothesis, trust propensity did not reliably predict truth judgments, *b* = −0.02, *SE* = 0.03, 95% CI [−0.06, 0.03], *z* = −0.64, *p* = 0.52, odds ratio (*OR*) = 0.98. Social trust was also not a significant predictor of truth judgments, *b* = −0.04, *SE* = 0.03, 95% CI [−0.10, 0.02], *z* = −1.31, *p* = 0.19, *OR* = 0.96.

As a robustness check, we also examined a multiple regression model that included both trust propensity and social trust as predictors. The effects remained non-significant: trust propensity: *b* = 0.09, *SE* = 0.08, 95% *CI* [−0.07, 0.25], *z* = 1.09, *p* = 0.28, *OR* = 1.10; social trust: *b* = 0.07, *SE* = 0.11, 95% *CI* [−0.16, 0.29], *z* = 0.57, *p* = 0.57, *OR* = 1.07.

**Non-preregistered: Bayesian analysis.** We complemented these analyses with Bayesian testing to quantify the evidence for the null hypothesis. We interpreted the Bayes factors based on Kass and Raftery’s (1995) suggested thresholds. Bayesian analyses, using weakly informative normal priors [*normal*(0, 10)] for regression coefficients and weakly informative t priors [*t*(3, 0, 10)] for the intercept, were conducted separately for trust propensity and social trust. Both analyses supported the null hypothesis, indicating that neither variable credibly predicts truth judgments. Specifically, the Bayes factor for the effect of trust propensity suggested that the null hypothesis was approximately 1,235 times more likely than the alternative (*BF*₁₀ = 0.00081, *BF*₀₁ = 1,235). Similarly, the Bayes factor for the effect of social trust provided strong support for the null model (*BF*₀₁ = 114.94).

To ensure the robustness of these findings, we also conducted a sensitivity analysis using different priors. For each predictor, we tested a weakly informative prior *normal(0,10)*, a more restrictive prior *normal(0,0.1)*, and a moderately informative prior *normal(0,1)* (see Gelman et al., 2013). In all analyses, the 95% credibility intervals for both trust propensity and social trust included zero. Bayesian hypothesis tests for both trust propensity and social trust showed very strong to decisive evidence for the null model over the alternative, with all BF₀₁ > 1,000 (corresponding to BF₁₀ < 0.001).

**Exploratory: Effect of deception base rate belief**. We finally explored the relationship between deception base rate belief and truth judgments using a similar mixed-effects model. The analysis showed no significant predictive effect of deception base rate belief, *b* = 0.002, *SE* = 0.002, 95% CI [1.00, 1.01], *z* = 1.03, *p* = 0.30, OR = 1.00.

### Discussion

Study 1 aimed to investigate whether trust propensity and social trust influence truth judgments. Given that previous research has demonstrated the impact of trust propensity on various behaviors, it seemed plausible that individuals with a greater general tendency to trust others and to think they are trustworthy would also be more likely to judge ambiguous statements as true. However, contrary to our hypothesis, the results did not support this assumption. Although a significant correlation between deception base rate belief and trust propensity/social trust was found, neither predicted truth judgments. Bayesian analysis provided substantial evidence for these null effects.

One possible explanation for the null effect lies in the fundamental nature of trust itself. Theoretical perspectives on trust emphasize that trust always involves an agent—a trustee who is the target of trust (Baer et al., 2018; Mayer et al., 1995). In contrast, the truth judgments examined in the present study were made in isolation, meaning that participants assessed statements without any accompanying source or contextual cues. In real-world contexts, truth judgments often involve implicit or explicit cues about the source of information (e.g., a speaker, an institution, or a media outlet), which may activate individual differences in trust propensity. Study 2 aimed to address this limitation by incorporating the social context in which truth judgments are made.

## Study 2

The goal of Study 2 was to conceptually replicate the design of Study 1 while integrating more social cues. The study design, materials, sample size, and exclusion rules were pre-registered: https://aspredicted.org/RVV_3DV.

### Methods

#### Participants and design

As in Study 1, participants were recruited from Prolific for a two-part study. Based on our observations from Study 1, we expected a lower dropout rate and thus oversampled by only 15%. We otherwise maintained the same power consideration and the same exclusion criteria and recruited 290 participants at T1 (aiming for a final sample size of 250). Two hundred eighty-eight participants completed the T1 questionnaire. However, only 193 of them completed both parts of the study. Three participants failed both attention checks and were excluded from analysis. The final sample consisted of 190 participants, including 101 men, 86 women, and three individuals who identified as non-binary or other (*M_age_* = 43.77, *SD* = 25.25). The study design was similar to Study 1: we measured trust propensity/social trust and deception base rate belief at T1. Two days later (T2), participants were asked to rate the same 37 statements as in Study 1, in a revised context integrating more social cues (see below).

### Materials

**Trust Propensity/Social Trust (T1).** We used the same 11 items from Study 1 to measure trust propensity and social trust. All items were rated on a 7-point Likert scale (1 = Strongly disagree to 7 = Strongly agree).

**Deception Base Rate Belief (T1).** Similar to Study 1, a slider ranging from 0 to 100 percent was used to assess deception base rate belief. The item was worded as follows: “In life, people might try to lie to you if they get the chance, or they could try to be honest. What percentage of people do you expect to lie to you in any given interaction?” (*M* = 36.84, *SD* = 20.56).

**Truth Judgments (T2).** We made three significant modifications to the materials to address the limitations identified in Study 1. First, participants were led to believe that the statements they would judge had been written by previous participants. This approach aimed to emphasize that the statements came from individuals, thereby providing a human source for the judgments. The second change involved the way the truth judgments were presented: they now appeared in speech bubbles, each visually connected to an icon of an alleged participant with its participant number. The last change involved the labels of the binary decision made by the participants, which were adapted from “true” versus “false” to “The participant is telling me the truth” versus “lying to me.” This labeling has implications for the interpretation of the findings: it means we are more directly measuring whether a person is intentionally making a false statement (i.e., lying) as opposed to, for example, being wrong or poorly informed. We intentionally decided to focus on the former to bring the measure closer to the deception literature (Levine, 2024).

Participants judged an average of *M* = 18.55 (*SD* = 4.85) statements as true. No statement was judged as either false or true by all participants, and average truth ratings per statement ranged from *M* = 0.28 to *M* = 0.72.

### Results

#### Factor analysis on the trust propensity/social trust measure

Based on the findings from Study 1, we conducted a confirmatory factor analysis (CFA) to test whether the two-factor structure of trust propensity and social trust would replicate. To ensure full comparability with Study 1 for any subsequent analyses, all 11 items were retained in the follow-up studies. However, the present analysis focuses on the subset of nine items that best capture the underlying two-factor structure, consistent with Study 1. The latent variables in the model were defined as trust propensity and social trust, which were again measured with five and four items, respectively. The model demonstrated a very good fit to the data, *χ*^2^(26) = 51.14, *p* = 0.002, *χ*^2^/df = 1.97, CFI = 0.975, TLI = 0.965, RMSEA = 0.077, 90% CI [0.045, 0.108], SRMR = 0.037. All items significantly loaded onto their respective factors, with standardized loadings ranging from 0.50 to 0.92, *p_s_* < 0.001. We thus computed two separate mean scores for trust propensity (*M* = 4.58, *SD* = 1.12; *α* = 0.91) and social trust (*M* = 3.73, *SD* = 1.05; *α* = 0.74).

Zero-order correlations show that social trust and trust propensity were positively correlated, *r* = 0.64, *p* < 0.001, 95% CI [0.56, 0.71]. Social trust was also negatively related to deception base rate belief, *r* = −0.50, *p* < 0.001, 95% CI [−0.65, −0.49], indicating that individuals who believe others are more likely to lie in general report lower social trust. Trust propensity was similarly related to deception base rate belief, *r* = −0.49, *p* < 0.001, 95% CI [−0.62, −0.43].

#### Testing the relationship between trust propensity/social trust and truth judgments

**Preregistered analysis.** We again tested the relationship between trust propensity/social trust and truth judgments using mixed-effects models (binomial distribution; evaluating one predictor at a time). Contrary to our hypothesis, trust propensity did not reliably predict truth judgments, *b* = 0.003, *SE* = 0.028, 95% *CI* [−0.05, 0.05], *z* = 0.11, *p* = 0.91, *OR* = 1.00. Social trust was also not a significant predictor of truth judgments, *b* = −0.05, *SE* = 0.03, 95% *CI* [−0.11, 0.01], *z* = −1.62, *p* = 0.11, *OR* = 0.95.

As a robustness test, we also examined a multiple regression model that included both trust propensity and social trust. The effects remained consistently non-significant: trust propensity: *b* = 0.002, *SE* = 0.083, 95% *CI* [−0.16, 0.16], *z* = 0.01, *p* = 0.99, *OR* = 1.00; social trust: *b* = −0.18, *SE* = 0.11, 95% *CI* [−0.40, 0.03], *z* = −1.36, *p* = 0.18, *OR* = 0.86.

**Non-preregistered: Bayesian analysis.** We supplemented these analyses with Bayesian testing to measure the evidence for the null hypothesis (testing separate models for trust propensity and social trust), using weakly informative normal priors [normal(0, 10)] for regression coefficients and weakly informative t priors [t(3, 0, 10)] for the intercept. Bayesian analyses supported the null hypothesis that neither trust propensity nor social trust credibly predicts truth judgments. Specifically, the Bayes factor for the effect of trust propensity suggested that the null hypothesis was approximately 787 times more likely than the alternative (BF₁₀ = 0.0013, BF₀₁ = 787.40). Similarly, the Bayes factor for the effect of social trust provided very strong evidence for the null model (BF₀₁ = 310.56).

We then conducted a sensitivity analysis using the same three prior specifications as in Study 1: a weakly informative prior *normal (0,10)*, a restrictive prior *normal (0,0.1)*, and a moderately informative prior *normal (0,1)* (Gelman et al., 2013). These indicated more variation in the findings depending on the prior specification than emerged in Study 1.

Regarding trust propensity, all 95% credibility intervals included zero across models, and Bayesian hypothesis testing supported the null hypothesis. Specifically, Bayes factors ranged from BF₀₁ = 9.17 (restrictive prior) to BF₀₁ = 2043.47 (medium prior) to BF₀₁ = 614.81 (weak prior), indicating moderate to strong and very strong evidence for the null model (Kass and Raftery, 1995).

Regarding social trust, the results were more mixed. Under weak and moderate priors, the null model was strongly favored (BF₀₁ = 265.85 and BF₀₁ = 46.46, respectively), whereas the restrictive prior produced a Bayes factor close to 1 (BF₀₁ = 1.05), indicating no clear preference between the null and alternative hypotheses. This suggests that the evidence for or against an effect of social trust on truth judgments depends on the prior assumptions, and that under stricter assumptions, the data do not clearly support either the presence or absence of an effect.

**Exploratory: Effect of deception base rate belief.** We finally conducted a mixed-effects model to explore the relationship between deception base rate belief and truth judgments. Results revealed no significant association between the two constructs, *b* = −0.0002, *SE* = 0.003, 95% CI [0.99, 1.006], *z* = −0.07, *p* = 0.94, OR = 1.00.

### Discussion

Study 2 aimed to address the limitations of Study 1 by incorporating a social context into the truth judgments made by the participants, introducing speech bubbles and attributing statements to previous participants. Emphasizing that the statement to be judged is provided by another human creates a setting that more closely resembles real interpersonal interactions, potentially activating trust propensity. In spite of these changes, the results remained consistent with Study 1: although a significant correlation between deception base rate belief and trust propensity/social trust emerged again, neither predicted truth judgments, a null finding further supported by additional Bayesian analyses.

Reflecting on the design of Study 2, we concluded that while the social context should have been adequately incorporated, the perceived importance of making correct truth judgments may still have been somewhat low. Indeed, both Studies 1 and 2 involved statements that represented truths or potential lies about the world, with no strong personal connection to the truth judgments. Given that trust propensity is a deeply personal factor, it is likely to influence behavior more in situations of personal importance and ambiguity.

We designed Study 3 to address these concerns by utilizing scenarios involving legal decisions. In these situations, higher personal stakes are expected to make trust propensity more relevant, providing a more meaningful context for examining its impact on truth judgments.

## Study 3

Study 3 adopted a courtroom framework inspired by the jury system used, for example, in the United States. This design simulated a legal setting where participants assumed the role of jurors and were tasked with making critical decisions, specifically assessing whether statements made by witnesses were more likely to be true or false. The study design, materials, sample size, and exclusion rules were pre-registered: https://aspredicted.org/V89_S36.

### Methods

#### Participants and design

To ensure that participants would be familiar with the US jury system, we recruited US participants from Prolific for a two-part study. We determined the sample size based on Brysbaert and Stevens’ (2018) suggestion that at least 1,600 data points are needed for such repeated measurements within a mixed model (i.e., statements as a repeated measure). To prevent excessive length and participant fatigue, we balanced the number of participants against the number of statements and decided to recruit 212 participants, who were asked to rate 10 statements each. To account for potential exclusions and dropouts between T1 and T2, we oversampled by 15% and recruited 250 participants at T1. Overall, 224 participants completed both T1 and T2, but in accordance with our preregistered exclusion criteria, we excluded one participant who failed both attention checks from analysis. Our final sample consists of 223 respondents (116 men, 101 women, and six non-binary or other); *M_age_* = 42.18, *SD* = 14.29.

The first part of this study followed the procedure of Studies 1 and 2, with the added assessment of different types of deception-based rate belief. At T2, participants were asked to rate 10 statements, presented in random order, by indicating whether they believed the witness was telling the truth or lying.

### Materials

**Trust Propensity/Social Trust (T1).** We used the same 11 items as in previous studies to measure trust propensity/social trust. All items were rated on a 7-point Likert scale (1 = Strongly disagree to 7 = Strongly agree).

**Deception Base Rate Belief (T1).** In Study 3, we included five questions measuring deception base rate beliefs in various social groups: general, family, friends, colleagues, and court (i.e., “In life, people might try to lie to you if they get a chance, or they could try to be honest. What’s the percentage of people you would expect to lie to you in any given interaction?” and “In any given court case, it is commonly acknowledged that witnesses may provide misleading or false information, often due to various motivations or pressures. What’s the percentage of people you would expect to lie when testifying in court?”; % scale from 0 to 100). Answers showed notable variation between social groups. The item measuring the general likelihood of people lying in any given interaction (replicating Studies 1 and 2) resulted in a relatively higher average response (*M* = 40.91, *SD* = 22.81). Deception base rate belief pertaining to colleagues was just as high (*M* = 40.76, *SD* = 25.29). In contrast, rates pertaining to close others were lower (family: *M* = 30.47, *SD* = 24.61; friends: *M* = 28.66, *SD* = 22.74). Finally, and most central to the present analysis, deception base rate beliefs within court cases were moderate (*M* = 31.55, *SD* = 24.01).

**Truth Judgments (T2).** We developed statements ourselves based on real cases and witness statements from US court materials (see Supplementary material SM2). We pretested 60 statements in a pilot study (*N* = 91) and selected the 10 most ambiguous in terms of perceived believability. These statements were presented as alleged court testimonies from witnesses; specifically, they were introduced as “reflecting testimonies that jury members may encounter in court hearings,” focusing on theft and traffic offenses. An example statement about theft is “During my lunch break outside the store, I saw the suspect running through the rain and stealing the smartphone out of someone’s bag. I guess the bag belonged to a customer.” An example of a traffic offense statement is: “The man disregarded the right of way and caused an accident at the intersection. I saw it all unfold from the sidewalk across the street while walking home from work.” Participants were reminded to consider the possibility that witnesses could be either truthful or deceptive and to evaluate each statement separately from their judgments of previous statements. Each participant rated 10 statements in a randomized order on a binary scale (1 = I feel the witness making this statement is telling the truth; 0 = I feel the witness making this statement is lying).

Participants rated an average of *M* = 6.65 (*SD* = 1.96) statements as true. No statement was judged as either false or true by all participants, and average truth ratings per statement ranged from *M* = 0.52 to *M* = 0.86.

### Results

#### Factor analysis on the trust propensity/social trust measure

Again, we conducted a CFA to examine the two-factor structure of trust propensity (five items) and social trust (four items). To ensure full comparability with Studies 1 and 2 for any subsequent analyses, all 11 items were included in the questionnaire. However, the current analysis focuses on the subset of nine items that best capture the underlying two-factor structure, consistent with Studies 1 and 2. The model again demonstrated a good fit to the data: *χ*^2^(26) = 55.40, *p* = 0.001, *χ*^2^/df = 2.13, CFI = 0.980, TLI = 0.972, RMSEA = 0.073, 90% CI [0.046, 0.100], SRMR = 0.029. All items loaded on the expected factor (standardized loadings ranged from 0.54 to 0.92, *p_s_* < 0.001). We thus computed two separate mean scores for trust propensity (*M* = 4.20, *SD* = 1.60; *α* = 0.94) and social trust (*M* = 3.49, *SD* = 1.31; *α* = 0.83). Social trust was positively correlated with trust propensity, *r* = 0.73, 95% CI [0.66, 0.73], *p* < 0.001.

#### Testing the relationship between trust propensity/social trust and deception base rate beliefs

We computed Pearson’s correlations between mean trust propensity/social trust and the five different deception base rate beliefs (i.e., general public, family, friends, colleagues, and individuals in court). Results revealed negative correlations overall, which were stronger for deception base rate beliefs related to people in general or more distant others compared to close others (mainly family; see Table 2).

**Table 2:** Correlations between the five types of deception, base rate belief, trust propensity (TP), and social trust (ST) in Study 3.

Type of deception-based rate belief	*r* (TP)	95% CI (TP)	*r* (ST)	95% CI (ST)
General	−0.48***	[−0.58, −0.37]	−0.64***	[−0.71, −0.55]
Colleagues	−0.40***	[−0.50, −0.28]	−0.56***	[−0.64, −0.46]
Court	−0.38***	[−0.49, −0.26]	−0.36***	[−0.47, −0.23]
Friends	−0.32***	[−0.43, −0.19]	−0.41***	[−0.51, −0.29]
Family	−0.23***	[−0.36, −0.10]	−0.35***	[−0.46, −0.22]

TP, Trust Propensity; ST, Social Trust. *** All correlations are significant at *p* < 0.001.

#### Testing the relationship between trust propensity/social trust and truth judgments

As before, we examined the relationship between trust propensity/social trust and truth judgments using mixed-effects models (binomial distribution; testing one predictor at a time). Contrary to our hypothesis, trust propensity did not reliably predict truth judgments, *b* = −0.003, *SE* = 0.034, 95% CI [−0.07, 0.06], *z* = −0.09, *p* = 0.93, OR = 1.00. Social trust was also not a significant predictor of truth judgments, *b* = −0.052, *SE* = 0.043, 95% CI [−0.13, 0.03], *z* = −1.23, *p* = 0.22, OR = 0.95.

As a robustness test, we also examined a multiple regression model that included both trust propensity and social trust. The effects remained nonsignificant, although social trust was approaching the 0.05 alpha threshold; trust propensity: *b* = −0.045, SE = 0.096, 95% CI [−0.23, 0.14], *z* = −0.47, *p* = 0.64, OR = 0.96; social trust: *b* = −0.27, SE = 0.14, 95% CI [−0.55, 0.00], *z* = −1.92, *p* = 0.055, OR = 0.76.

**Non-preregistered: Bayesian analysis.** We complemented these analyses with Bayesian testing to quantify the amount of evidence for the null (with separate tests for trust propensity and social trust), using weakly informative normal priors [normal(0, 10)] for regression coefficients and weakly informative t priors [t(3, 0, 10)] for the intercept. Bayesian analyses supported the null hypothesis that neither trust propensity nor social trust credibly predicts truth judgments: for trust propensity, BF₁₀ = 0.008, suggesting that the null hypothesis is approximately 122 times more likely than the alternative (BF₀₁ = 121.78); for social trust, BF₁₀ = 0.011 or BF₀₁ = 94.70.

To assess the robustness of our findings, we conducted a Bayesian sensitivity analysis using three different prior specifications, following the same approach as in Studies 1–2: a weakly informative prior *normal(0, 10)*, a moderately informative prior *normal(0, 1)*, and a restrictive prior *normal(0, 0.1)* (Gelman et al., 2013).

For trust propensity, across all models, the 95% credibility intervals for the regression coefficients included zero. Bayesian model comparisons consistently favored the null model over the model including trust propensity as a predictor. Specifically, Bayes factors indicated moderate to strong evidence in favor of the null model, with BF₀₁ = 432.90 for the model with a weak prior, BF₀₁ = 88.91 for the model with a moderate prior, and BF₀₁ = 9.88 for the model with a restrictive prior.

Regarding social trust, the results also supported the null hypothesis, although the strength of evidence varied depending on the priors. All 95% credibility intervals included zero. The Bayes factors were BF₀₁ = 111.83 under the weak prior (strong evidence for the null), BF₀₁ = 12.67 under the moderate prior (positive evidence for the null), and BF₀₁ = 1.98 under the restrictive prior (anecdotal evidence for the null; Kass and Raftery, 1995).

**Exploratory: Effect of deception base rate belief.** We conducted two generalized linear mixed-effects models (GLMMs) with logit link functions to examine the relationship between deception base rate beliefs and truth judgments. In a direct replication of Studies 1 and 2, the first model examined the relationship between participants’ generalized deception base rate beliefs and their truth judgments. Results indicated no significant association, *b* = 0.002, *SE* = 0.002, 95% CI [−0.002, 0.006], *z* = 0.69, *p* = 0.49, OR = 1.00. The second model tested court-specific deception base rate beliefs. Again, no significant effect was found, *b* = −0.002, *SE* = 0.002, CI [−0.006, 0.002], *z* = −0.82, *p* = 0.41, OR = 0.99.

### Discussion

Study 3 made a final attempt to identify a relationship between trust propensity, social trust, and truth judgments. To achieve this, we introduced a courtroom framework to simulate a legal decision-making environment, in which participants acted as jury members. This design was intended to enhance the social and contextual relevance of truth judgments.

Despite these efforts to create a more realistic and relevant setting, the results from Study 3 were consistent with those from Studies 1 and 2, indicating null effects, which were further substantiated by Bayes factors supporting the null hypothesis. This suggests that, even within a high-stakes legal context, trust propensity did not influence participants’ assessments of witness testimonies.

As a robustness check, we also examined a multiple regression model that included both trust propensity and social trust as predictors. In this model, the effect of social trust approached significance but in a direction opposite to expectations. The trend would indicate that, when controlling for trust propensity, the variance that is unique to social trust is actually related to a greater likelihood of rating statements as *lies*. It is important to note, however, that such a suppression effect only emerged in this study, that it still did not meet the threshold for statistical significance, and that the mean scores of social trust and trust propensity were highly correlated. This specific finding may thus reflect sampling variability or multicollinearity between the two predictors, and we are cautious not to overinterpret it.

As in Study 2, we implemented a binary truth judgment task, in which participants classified each statement either as the witness telling the truth or lying. As briefly highlighted above, this operationalization was deliberately chosen to strengthen the social dimension of the task by emphasizing intentional deception as a central concern in legal and interpersonal contexts. However, this design also constrains interpretability: by using “lying” as the sole option for a false judgment, the task precludes the possibility that a statement may be false without involving intentional deception—for instance, due to misinformation, faulty reasoning, or honest error. Thus, while the binary framing reinforced the interpersonal relevance of the task, it may have conflated epistemic accuracy with moral attribution.

## General discussion

Understanding truth judgments requires considering individuals’ underlying assumptions about the prevalence of truth and deception. We conducted three preregistered studies to examine whether trust propensity (and social trust) positively influenced truth judgments. Our starting assumption was that people who believe that others are generally more trustworthy may be more inclined to believe their statements, which could lead to a biased tendency toward more positive truth judgments among individuals with higher trust propensity (or social trust).

### Trust propensity and truth judgments are unrelated

Contrary to our hypothesis, the three studies consistently indicate that neither trust propensity nor social trust significantly influences the likelihood of perceiving different statements as true. This finding was robust across studies employing distinct paradigms, ranging from context-free truth judgments to social and legal contexts. Bayesian analyses further provided substantial evidence in favor of the null hypothesis, emphasizing that trust propensity and social trust do *not* play a meaningful role in determining whether individuals judge statements as true or false, or as truth or lie.

At first glance, the absence of an association between trust propensity and social trust with truth judgments may seem counterintuitive. Given that trust is fundamental to interpersonal interactions (Mayer et al., 1995) and plays a role in shaping cooperation, risk-taking, and credibility assessments (Colquitt et al., 2007; Yamagishi et al., 2015), it appeared reasonable to expect that individuals with a higher trust propensity would be more inclined to accept information as truthful. One possible explanation for the present null findings is that truth judgments depend more strongly on cognitive mechanisms, such as fluency and memory consistency, rather than stable personality traits.

Our findings also suggest that, alone, contextualization may not be sufficient to elicit trust-based reasoning in truth judgments. While we attempted to increase social salience in Studies 2 and 3 by attributing statements to human sources and embedding them in legal contexts, these manipulations may not have sufficiently engaged trust-based reasoning. The courtroom paradigm, for instance, might have activated skepticism rather than trust, given the inherent adversarial nature of legal decision-making (Saks and Spellman, 2016).

In an exploratory manner, we also measured deception base rate beliefs, which are one’s general tendency to believe that people tell the truth or lie. Trust propensity and deception base rate beliefs were significantly correlated across all studies, suggesting that individuals who generally trust others also tend to assume a higher prevalence of truthful statements by others. However, deception base rate beliefs were also unrelated to the actual ratings of judgments as “true” or “false” (Study 1), ratings that “the participant is telling me the truth” or “lying to me” (Study 2), or feelings that “the witness making this statement is telling the truth” or “is lying” (Study 3).

While theories on truth judgments highlight base rate beliefs as an influential factor (Brashier and Marsh, 2020), such beliefs have rarely been explicitly measured in prior research. In contrast, our study incorporated a direct measurement approach. The results suggest that deception base rate beliefs may play a weaker role in truth judgments than previously assumed, or that our measure did not accurately capture these beliefs. Some accounts of base rate effects propose that such beliefs operate at a more implicit or automatic level (Levine, 2018a), whereas our studies relied on self-report measures to assess participants’ perceived frequency of deception. This raises the possibility that reported beliefs do not fully reflect the cognitive processes underlying truth judgments.

This conclusion—that base rate beliefs may play a limited role in truth judgments—is further supported by empirical evidence from communication studies, and more specifically from Park and Levine (2015), who argue that individuals are largely insensitive to sender veracity. According to their Park-Levine Model (PLM), deception detection accuracy is primarily driven by the actual base rate of truths and lies, not by individuals’ awareness of these rates (Park and Levine, 2001). People tend to exhibit a robust truth bias, consistently judging messages as honest regardless of actual veracity. As a result, changes in the proportion of truthful versus deceptive messages (i.e., the base rate) affect overall accuracy but not participants’ beliefs about whether a given message is true or false. Their findings, replicated across multiple interactive and non-interactive designs, show that receivers seldom classify deceptive messages as such—even when given cues or explicit incentives to detect deception. Thus, the PLM undermines the idea that individuals actively incorporate base rate beliefs into their veracity judgments, instead positioning these beliefs as structural features of the communication environment rather than as consciously used inferential tools.

### Distinguishing trust propensity and social trust

Beyond the question of whether trust propensity influences truth judgments, our findings contribute to a more precise conceptualization of the construct itself. While both trust propensity and social trust have been extensively studied, the distinction between them has often been blurred at the measurement level. The present study makes a novel contribution by systematically disentangling which items capture each construct and by directly comparing them within the same framework. Our factor analysis revealed a two-factor structure that distinguished between trust propensity, which reflects a personal tendency to trust others (“I am trusting”), and social trust, which captures generalized expectations about the trustworthiness of others (“Others are trustworthy”). While this distinction aligns with theoretical definitions, it has typically been overlooked in empirical studies. Future research may be well advised to take this differentiation into consideration and ensure that studies measuring the early stages of trust explicitly define which construct is being examined.

### Limitations, future directions, and conclusion

The present studies were conducted in highly controlled and artificial settings designed to isolate the potential effects of trust propensity and social trust on truth judgments. While this approach allows for internal validity and systematic comparison, it necessarily limits the ecological validity of the findings. Future research should aim to examine truth judgments in real-world contexts, such as real courtroom settings or media consumption. These contexts typically involve uncertainty and higher stakes, which can amplify the role of individual traits, such as trust propensity and social trust.

A second limitation concerns the specificity of the trait-focused approach. We focused on trust-related dispositions due to their conceptual proximity to truth judgments. In doing so, we did not examine alternative explanatory mechanisms—such as fluency, negativity bias, or general state-based influences—that may better account for variance in perceived truth. Future research should expand the scope by including such factors and testing their relative explanatory power. In addition, other trust-related dispositions should be investigated. For example, a more cognitive and motivational approach to trust could be integrated by exploring the role of “trusting stance” (McKnight and Chervany, 2001), which describes a person’s strategic or principle-driven willingness to trust, even without strong expectations of reciprocity. Incorporating this motivational dimension would enable us to capture a more comprehensive picture of early trust decisions, particularly in situations characterized by uncertainty or moral ambiguity.

Moreover, although we attempted to capture base rate sensitivity through a direct question, there is currently no validated measure of base rate assumptions in the context of truth judgments. Our single-item measure provides an initial attempt, but future studies should develop and validate more nuanced tools to assess this construct more reliably.

Finally, although our study meaningfully differentiates between trust propensity and social trust, we recognize that the measurement of these constructs in prior research has often been confounded. While we addressed this issue by factor analyzing the items and demonstrating distinct underlying dimensions, future studies should further refine and validate these measurements, especially in applied settings.

In conclusion, our findings refine prior theories (e.g., Truth-Default Theory, Levine, 2018b, 2020) by showing that, across different social contexts, trust propensity does *not* bias truth judgments in either direction. Bayesian analyses consistently support the null hypothesis, suggesting that truth judgments are not systematically shaped by dispositional trust, even in situations where intuitive, “gut-feeling” decisions are required. Moreover, we establish a clear conceptual and empirical distinction between trust propensity and social trust, two constructs that have often been conflated in prior work. Together, these insights refine the theoretical landscape on dispositional influences in truth judgments and clarify the boundaries of when and how trust traits matter.

## Data Availability

The datasets presented in this study can be found in online repositories. The names of the repository/repositories and accession number(s) can be found in the article/Supplementary material.
