# Supplementary material for: Distrusting minds, skeptical judgments? No evidence for a trust-truth link
Source: Front Psychol. 2025 Sep 30;16:1626047. doi: 10.3389/fpsyg.2025.1626047 (PMC12518263; doi:10.3389/fpsyg.2025.1626047)
Supplement: Supplementary file 1 [file Supplementary_file_1.docx]

**Supplemental Material: Distrusting Minds, Skeptical Judgments? No Evidence for a Trust-Truth Link**

**Table SM1.** *Overview of the statements used in Study 1 and Study 2*

| **Truth judgment statements** |  |
| --- | --- |
| Drinking alcohol decreases your core body temperature. | |
| Vitamin C is an ineffective treatment for a cold. | |
| Blackberries and raspberries belong to the rose family. | |
| Exposing yourself to extreme cold temperatures regularly can increase resistance to cold-related illnesses. | |
| All states in Oceania have water borders only. | |
| The Cocos islands belong to Indonesia. | |
| A baseball team has 9 players. | |
| Flowers and plants are the topselling fairtrade products. | |
| Some turtles can breathe through their anus. | |
| The longest earthworm in the world is 6.7 meters long. | |
| Some people have a natural immunity to HIV. | |
| There is a lake in Tanzania that is deadly to all species who touch its water. | |
| The first windmills were in Persia. | |
| The name of the Russian space station MIR means peace. | |
| The rainiest place on earth is the summit of Mount Waialeale in Hawaii. | |
| A galactic year is approximately 2500 normal years. | |
| The Lama temple is located in Shanghai. | |
| The Barringer crater is on the northern moon hemisphere. | |
| Socrates was the teacher of Alexander the Great. | |
| The tallest tree in the world is a spruce. | |
| The first heart-lung machine was used in the Netherlands. | |
| Volcanos can reach a maximum height of approximately 5000 meters. | |
| The most expensive color pigment is the genuine ultramarine. | |
| The longest time a chicken ever flew was 13 seconds. | |
| The banana is a berry, but the strawberry is not. | |
| The smallest bone in the human body is smaller than a grain of rice. | |
| The human body contains enough carbon to make 900 pencils. | |
| The sun makes up about 99.86% of the total mass of the solar system. | |
| In a group of 57 people, the likelihood that two share the same birth-day is higher than 90 percent. | |
| The entire electricity powering the Internet weighs as much as an apricot. | |
| Lightning kills about 10,000 people a year worldwide. | |
| Rabbits can see what’s behind their head without moving their heads. | |
| Mistletoe has no scent. | |
| New Yorkers bite 10 times more people than sharks do worldwide each year. | |
| Sharks can close their retinas. | |
| Human fingers have no muscles. | |
| Measured from west to east, Australia is wider than the moon’s diameter. | |

**Table SM2.** *Overview of the truth judgment statements presented in Study 3*

| **Truth judgment statements** |  |
| --- | --- |
| "I remember seeing her tampering with the lock. She snatched the necklace and dashed towards the exit with a smug smile on her face. She was wearing a thick winter jacket." | |
| "It was a usual busy Saturday afternoon. I was browsing through the aisles when I suddenly heard a rustle. Peeking around the corner I witnessed her slipping the watch into her coat before running.” | |
| "During my lunch break outside the store, I spotted the suspect running through the rain and stealing the smartphone from someone's bag. I’m guessing the bag belonged to a customer." | |
| "The suspect seemed nervous when he rushed past me. Since he is very tall, he caught my eye. And that’s why I caught him stealing an expensive pullover." | |
| "Usually these types of things never happen, except on that Sunday when I saw the man in the city center running into the crowd and stealing the purse of that young lady." | |
| "The luxurious mall close to my home is a place I like strolling through in peace, but on that particular Monday there was a lot of commotion as that woman ran past me with three boxes of stolen perfumes." | |
| "Late at night, I was taking my dog out for a walk. I then observed the woman driving an old beat-up car and running a red light while texting and driving." | |
| "The man disregarded the right of way and caused an accident at the intersection. I saw it all unfold from the sidewalk across the street while walking home from work." | |
| "On a beautiful day outside, a woman drew my attention for driving well below the speed limit while being on the phone, forcing other drivers to adjust their own speed and ultimately causing an accident." | |
| “It was a rainy day and I saw the man in his expensive sports car texting behind the wheel right before colliding with another car at the intersection where he ran a red light." | |
